# Digital Patient-Reported Monitoring of Functional Recovery After Robot-Assisted Radical Prostatectomy in High-Risk Prostate Cancer Treated With or Without Perioperative Hormonal Therapy

**DOI:** 10.3390/diagnostics16172748

**Published:** 2026-08-27

**Authors:** Bogdan Adrian Buhas, Alessandro Uleri, Giorgio Calleris, Guilhem Roubaud, Marine Lesourd, Benjamin Pradère, Christophe Tollon, Damien Pouessel, Bernard Malavaud, Loïc Mourey, Guillaume Ploussard

**Affiliations:** 1 Urology Department, Groupe Hospitalier Diaconesses Croix Saint-Simon, 75020 Paris, France; 2 Urology Department, Clinique La Croix du Sud, 31130 Quint-Fonsegrives, France; 3 Urology Department, Hospital Universitari de Girona Doctor Josep Trueta, 17007 Girona, Spain; 4 Urology Clinic, Department of Surgical Sciences, University of Turin and Città della Salute e della Scienza, 10126 Turin, Italy; 5 Department of Medical Oncology, Institut Bergonié, 33000 Bordeaux, France; 6 Oncopole Claudius Regaud—IUCT-Oncopole, University Toulouse III—Paul Sabatier, 31062 Toulouse, France; 7 Toulouse Cancer Research Centre, 31100 Toulouse, France

**Keywords:** high-risk prostate cancer, robot-assisted radical prostatectomy, perioperative hormonal therapy, urinary continence, patient-reported outcome measures, digital health, remote monitoring, functional recovery

## Abstract

**Background/Objectives:** Perioperative androgen-deprivation therapy (ADT) and androgen-receptor pathway inhibitors (ARPIs) are increasingly used in high-risk prostate cancer, yet patient-centred functional-recovery data after radical prostatectomy are scarce and rarely standardized. We assessed whether a smartphone-based digital monitoring pathway could characterize social-continence recovery and capture differences associated with a perioperative hormonal therapy (PHT) strategy after robot-assisted radical prostatectomy with pelvic lymph-node dissection (RARP + LND). **Methods:** In a two-centre consecutive cohort (*n* = 98) of men with high-risk, non-metastatic prostate cancer aligned with SUGAR and PROTEUS criteria, we compared upfront surgery (standard of care [SOC], *n* = 72) with a planned PHT strategy (ADT or ARPI, *n* = 26); all patients were urinary-continent preoperatively. Clinical data were prospective; patient-reported and patient-experience measures were captured through a routine-care digital platform (Betty Coaching application) delivering an identical prehabilitation/rehabilitation pathway to both groups, with pre- and postoperative pelvic-floor muscle training performed by all patients. The primary endpoint was 6-month social continence (0–1 pad/day), assessed by clinician and patient reports as co-primary sources. Differences are reported as risk differences with 95% confidence intervals (CIs). **Results:** At 6 weeks, no statistically clear difference was observed (clinician 66.2% vs. 56.5%; risk difference 9.7 percentage points, 95% CI −12 to 32). At 6 months, social continence was lower with PHT (clinician 87.1% vs. 65.0%, difference 22.1 points, 95% CI 2.6 to 44.7; patient 81.3% vs. 58.3%, 22.9 points, 95% CI 2.4 to 43.9), converging by 12 months (96.9% vs. 95.0%). Analgesic-free rates were lower with PHT on postoperative days (POD) 7 and 10. **Conclusions:** Digital monitoring captured granular recovery trajectories and identified a mid-term social-continence signal associated with PHT, compatible with a transient delay in recovery. These hypothesis-generating findings support embedding standardized digital functional-outcome monitoring in perioperative trials.

## 1. Introduction

High-risk and locally advanced prostate cancer (PCa) is increasingly encountered in contemporary practice [1]. In men eligible for curative-intent local therapy, radical prostatectomy (RP), most commonly robot-assisted (RARP) with pelvic lymph-node dissection (LND), remains a cornerstone option, alone or within a multimodal strategy alongside radiotherapy and androgen-deprivation therapy (ADT) [2,3]. Nevertheless, a substantial proportion of men with high-risk disease develop biochemical recurrence, sustaining interest in perioperative treatment intensification [4,5,6].

Conventional neoadjuvant hormonal therapy improved pathological surrogates and margin status without a clear long-term oncological benefit [7,8,9,10], and is accordingly not recommended as the standard of care before RP in contemporary guidance [11]; yet it persists in real-world practice, and available patient-reported outcome measures (PROMs) suggest persistently worse hormonal quality-of-life (QoL) scores after surgery [12]. In parallel, modern androgen-receptor pathway inhibitors (ARPIs) have produced survival gains in non-metastatic castration-resistant [13], high-risk biochemically recurrent [14], and localized high-risk disease combined with radiotherapy [15], and contemporary programmes increasingly explore ARPI activity in earlier, localized disease [16,17,18,19].

This perioperative evidence remains dominated by pathological endpoints, with scarce and inconsistently reported patient-centred functional data [18,19]. Urinary continence is a dominant determinant of postoperative QoL, governed chiefly by urethral sphincter integrity and pelvic-floor dynamics [20,21,22]; reported continence rates after RP vary substantially with the definition and timing of assessment [23,24,25,26], and long-term functional outcomes remain a central survivorship concern [27,28,29]. The question is clinically pressing because androgen suppression impairs skeletal-muscle mass and performance and promotes sarcopenia [30,31,32], plausibly affecting sphincter and pelvic-floor function; whether, and over what time course, perioperative hormonal therapy (PHT) delays continence recovery remains insufficiently characterized to inform shared decision-making [12,18].

A central obstacle is measurement: conventional clinic-based assessment captures recovery sparsely and with variable definitions, and because recovery occurs continuously but is recorded only at scheduled assessments, interval censoring systematically overestimates recovery times [33]. Randomized evidence shows that systematic electronic PROMs monitoring improves symptom control, QoL, and survival in oncology [34,35,36,37]; eHealth follow-up applications are increasingly validated [38,39,40,41]; and machine-learning analyses of activity and recovery patterns are beginning to characterize post-treatment urinary incontinence [42]. The Betty Coaching application (https://app.betty.care/appstores) provides structured prehabilitation and rehabilitation content, checklists, remote monitoring, and systematic PROM/patient-reported experience measure (PREM) capture, with high compliance and accuracy in prior evaluations of robot-assisted onco-urological surgery [43,44,45,46,47].

We therefore used this routine-care digital pathway to characterize social-continence recovery and early postoperative outcomes after RARP + LND in men with high-risk PCa managed with upfront surgery versus a PHT strategy. We hypothesized that, if PHT transiently impairs muscle-dependent continence mechanisms, digital monitoring would identify a delayed mid-term continence signal resolving over longer follow-up. Because allocation was non-randomized, the analysis is hypothesis-generating and is not intended to establish a causal effect.

## 2. Materials and Methods

### 2.1. Study Design

We conducted a two-centre, consecutive comparative cohort study of men with high-risk PCa undergoing RARP with pelvic LND, using prospective institution-approved databases complemented by patient-reported measures collected through the Betty application, reported in accordance with the STROBE statement [48]. High-risk disease was defined according to the SUGAR (CCAFU-PR2) and PROTEUS inclusion criteria [49,50]. Eligible patients were consecutive men with non-metastatic (M0) high-risk or locally advanced PCa treated with curative-intent RARP + LND during the routine-use period of the digital pathway; all were urinary-continent (no pad use) at baseline clinical assessment, and salvage surgery and metastatic disease were excluded.

### 2.2. Study Population and Exposure Definition

Patients were categorized a priori by intended management: (i) upfront RARP + LND without planned perioperative hormonal therapy (standard of care, SOC); (ii) a planned PHT strategy administered 3–6 months before and 3–6 months after surgery, comprising ADT alone, an ARPI alone (darolutamide, apalutamide, or abiraterone acetate with prednisone), or both. Because postoperative treatment extended 3–6 months beyond surgery, the 6-month assessment coincided with, or shortly followed, ongoing hormonal exposure, whereas by 12 months, all patients were expected to have completed treatment.

Allocation was non-randomized, reflecting real-world multidisciplinary decision-making and trial-related pathways, and was therefore subject to confounding by indication. Because the treated cohort was modest and intentionally heterogeneous, mirroring real-world regimen selection [51], the exposure was prespecified at the level of the management strategy (any planned PHT versus none) rather than agent, class, or duration; regimen- or duration-stratified analyses would create subgroups too small for reliable inference.

### 2.3. Standardized Perioperative Pathway and Co-Interventions

Both groups followed an identical perioperative pathway at each centre: all patients performed structured pelvic-floor muscle training (PFMT) both before and after surgery, and prehabilitation and rehabilitation content, education, and recovery checklists were delivered uniformly through the Betty application, independent of allocation [43,45,46,47,52]. Because PFMT is a recognized determinant of early continence recovery [53,54,55,56], this uniformity reduces the likelihood that differential rehabilitation explains the observed outcomes.

### 2.4. Outcomes

The primary endpoint was social continence at 6 months after RARP + LND, defined as 0–1 (safety) pad/day, a definition widely used to permit comparison across studies [20,21,23], assessed identically in both groups by clinician- and patient-reported measures. Because all patients were continent preoperatively, postoperative rates reflect de novo, surgery-related incontinence. The two ascertainment methods were prespecified as complementary co-primary sources without formal hierarchy, each mitigating a different reporting bias; formal agreement analysis was not prespecified, and paired responses were not uniformly available. Strict pad-free continence (0 pads/day) was not available for all patients and was not analyzed separately.

Secondary endpoints were social continence at 6 weeks and 12 months; residual symptom burden among incontinent men (pads/day; incontinence-related QoL on a 0–10 visual analogue scale [VAS]); perioperative recovery and safety (length of stay [LoS], same-day discharge [SDD], complications, readmissions, reoperations, unplanned visits); daily postoperative pain and analgesic-use trajectories; and global satisfaction (VAS) [57].

### 2.5. Data Collection

Clinical and oncological data were captured prospectively in institution-approved databases; PROMs/PREMs were collected through the Betty application integrated into routine care [47,58]. Continence was assessed at scheduled, discrete time points (6 weeks, 6 and 12 months), whereas pain and analgesic use were captured daily early after surgery; app-level engagement metrics were not collected, and assessment-completion rates are reported instead.

### 2.6. Statistical Analysis

Continuous variables are summarized as means and categorical variables as counts with percentages over non-missing observations; missing assessments are reported per time point. Groups were compared using the χ^2^ or Fisher’s exact test and the Mann–Whitney *U* test. Principal comparisons are presented as risk differences with Newcombe 95% confidence intervals (CIs), so that magnitude and precision, rather than statistical significance alone, guide interpretation. Missing data were not imputed: in a cohort of this size, model-based sensitivity analyses would convey a precision that the data cannot support. Comparisons therefore use all available observations, in keeping with the study’s aim of characterizing what routine digital monitoring can capture in this population. No post hoc power calculations were performed; CI width conveys the uncertainty arising from sample size. Values of *p* are reported to one or two significant figures; *p* < 0.05 was considered significant. Analyses used SPSS v31.0 (IBM Corp., Armonk, NY, USA).

Disease aggressiveness and clinician and patient preference plausibly influence both PHT allocation and recovery, and androgen suppression may transiently impair muscle-dependent continence mechanisms [30,31]. All reported comparisons are unadjusted associations and are interpreted as such throughout.

## 3. Results

### 3.1. Study Population

Of 98 men, 72 underwent upfront RARP + LND and 26 received a PHT strategy; all were continent (no pad use) preoperatively and all performed pre- and postoperative PFMT (Table 1). Baseline demographics, disease burden, and antithrombotic use were comparable, including age, body mass index (BMI), prostate-specific antigen (PSA), prostate volume, and cN1 status (Table 1). The main imbalance was multiparametric MRI (mpMRI) suspicion, with PI-RADS 5 lesions in 80.8% of PHT versus 50.0% of SOC patients (*p* = 0.049); MRI stage (*p* = 0.5) and biopsy grade-group distribution (*p* = 0.1) were similar, as were intraoperative parameters (all *p* > 0.05) (Table 1).

### 3.2. Pathological Outcomes

Pathological outcomes were broadly comparable (Table 2): grade-group distribution (*p* = 0.5), pathological stage (*p* = 0.9), and pN1 rates (15.3% vs. 15.4%; *p* > 0.9). Nodal yield, positive margins (30.6% vs. 19.2%; *p* = 0.3), detectable postoperative PSA (22.2% vs. 7.7%; *p* = 0.1), and recurrence showed no statistically clear differences; because follow-up duration was not uniformly captured, recurrence data are descriptive only (Table 2).

### 3.3. Social-Continence Recovery

Completion of scheduled continence assessments (SOC vs. PHT) was as follows: 6 weeks—clinician 71/72 (99%) vs. 23/26 (88%), patient 72/72 (100%) vs. 21/26 (81%); 6 months—clinician 70/72 (97%) vs. 20/26 (77%), patient 64/72 (89%) vs. 24/26 (92%); 12 months—clinician 65/72 (90%) vs. 20/26 (77%). Patient-reported 12-month assessments were unavailable. Missingness reflected non-completion of the scheduled assessment; individual reasons were not systematically recorded.

**Figure 1:**
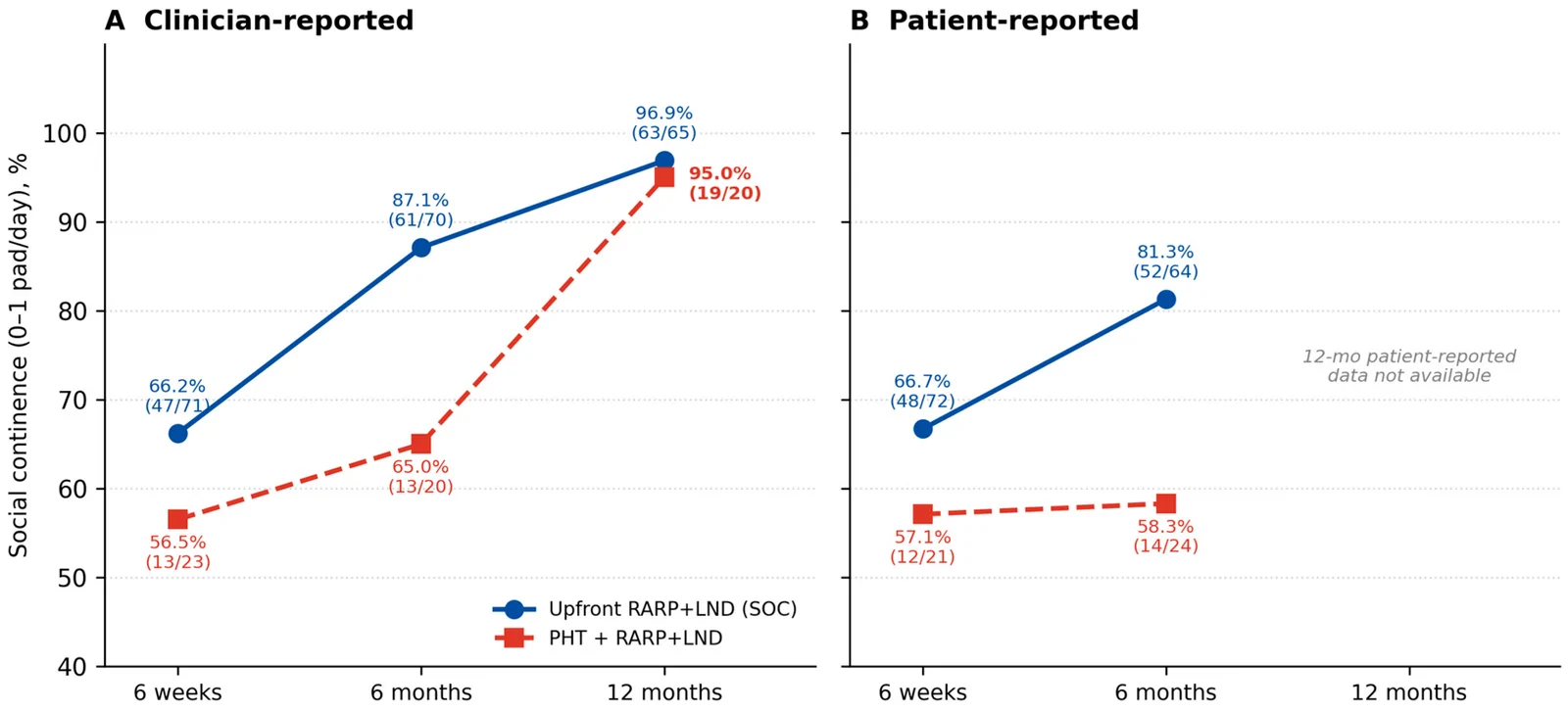
Social-continence (0-1 pad/day) trajectories after RARP + LND, by assessment source: (**A**) clinician-reported and (**B**) patient-reported. Points show the proportion continent with *n*/*N* below each time point. Solid line, upfront RARP + LND (SOC); dashed line, perioperative hormonal therapy (PHT). Patient-reported 12-month data were not available. The pattern, no clear difference at 6 weeks, lower with PHT at 6 months, and convergence at 12 months on clinician-reported assessment, is compatible with a transient delay in recovery; permanent impairment cannot be formally excluded, particularly on patient-reported measures.

At 6 weeks, no statistically clear difference was observed, although confidence intervals were wide (clinician 66.2% vs. 56.5%, risk difference 9.7 percentage points, 95% CI −12 to 32; patient 66.7% vs. 57.1%, 9.5 points, 95% CI −12 to 32) (Table 3, Figure 1).

At 6 months, social continence was lower with PHT by both sources: clinician-reported 87.1% (61/70) vs. 65.0% (13/20), risk difference 22.1 points (95% CI 2.6 to 44.7; *p* = 0.02); patient-reported 81.3% (52/64) vs. 58.3% (14/24), 22.9 points (95% CI 2.4 to 43.9; *p* = 0.03). The wide CIs indicate that, although a clinically meaningful difference is likely, its magnitude is imprecise. Among incontinent men, residual burden was comparable (0.8 vs. 1.1 pads/day, *p* = 0.7; QoL VAS 2.3 vs. 3.4, *p* = 0.6).

By 12 months, clinician-reported social continence was high in both groups (96.9% [63/65] vs. 95.0% [19/20], difference 1.9 points, 95% CI −7 to 21). This time course is compatible with a transient delay in social-continence recovery; because patient-reported 12-month data were unavailable, persistent impairment on patient-reported measures cannot be formally excluded (Figure 1).

### 3.4. Perioperative Recovery and Safety

LoS and SDD were similar (0.72 vs. 0.77 days, *p* = 0.7; 37.5% vs. 30.8%, *p* = 0.5). No statistically clear differences were observed in safety endpoints, but event numbers were small and estimates imprecise; numerically, complications and unplanned visits were higher with PHT (19.4% vs. 30.8%, risk difference −11.3 points, 95% CI −32 to 6; readmissions 5.6% vs. 11.5%; reoperations 1.4% vs. 7.7%; unplanned visits 4.2% vs. 15.4%, *p* = 0.06). Satisfaction was comparable (VAS 8.6 vs. 8.8) (Table 3).

### 3.5. Postoperative Pain and Analgesic Trajectories

**Figure 2:**
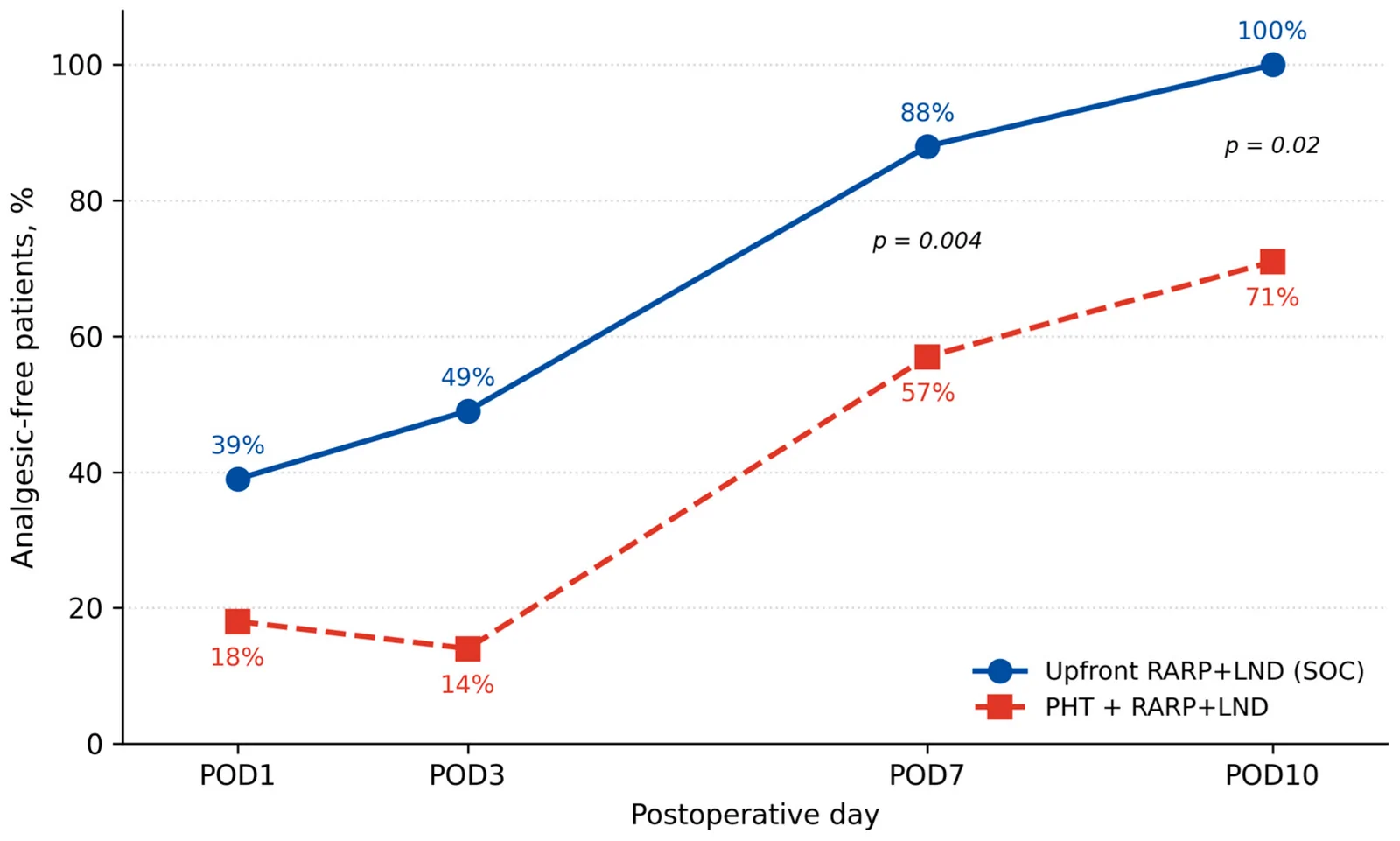
Proportion of analgesic-free patients by postoperative day (POD) after RARP + LND. Solid line, upfront RARP + LND (SOC); dashed line, perioperative hormonal therapy (PHT). Proportions are among patients responding on each day and are exploratory; daily denominators varied. Between-group *p*-values are shown for POD7 and POD10.

Daily monitoring captured analgesic use and pain (Table 4, Figure 2). Analgesic-free proportions were lower with PHT from postoperative day (POD) 1 (39% vs. 18%) and reached significance at POD7 (88% vs. 57%, *p* = 0.004) and POD10 (100% vs. 71%, *p* = 0.02); pain (VAS) was similar through POD7 but higher with PHT at POD10 (0.5 vs. 1.7, *p* < 0.001). Given daily variation in responders, these trajectories are exploratory [58].

## 4. Discussion

In this two-centre cohort of 98 consecutive men undergoing RARP + LND for high-risk PCa, a routine-care digital monitoring pathway captured functional-recovery trajectories and identified a delayed mid-term social-continence signal associated with PHT: no clear difference at 6 weeks, lower continence at 6 months by both clinician and patient reports, and similarly high 12-month rates on clinician-reported assessment. Daily capture additionally revealed more prolonged analgesic use with PHT. All findings are unadjusted associations from a non-randomized cohort. The contribution is methodological as much as clinical: it suggests that standardized, smartphone-based monitoring can support the detection of clinically meaningful, time-dependent differences that sparsely sampled clinic assessment would likely miss.

Functional recovery after RARP is usually assessed at few visits and with heterogeneous definitions [23], limiting sensitivity to transient effects. Two clarifications delimit our claim. First, the truly high-frequency (daily) data here concerned pain and analgesics; continence was assessed at scheduled, discrete anchors, so the pathway’s added value for continence lay in standardized, dual-source ascertainment rather than continuous measurement. Second, app-level engagement and usability metrics were not collected, so feasibility is supported only indirectly, through the reported assessment-completion rates and prior dedicated evaluations of the platform showing high adherence and accuracy [43,44,45,46,47]. Within the same ecosystem, the BETTY score predicts perioperative morbidity [59], and one-year outcomes after same-day-discharge RARP support digitally monitored outpatient pathways [60], and national claims-based benchmarking of early-adopter centres using the same pathway suggests favourable SDD and LoS profiles [61]. Our findings are consistent with randomized evidence that systematic electronic PROM monitoring improves patient-centred outcomes in oncology [34,35,36,38,62] and extend it by showing that such monitoring can discriminate between management strategies, supporting its use as a structured outcome-ascertainment instrument in comparative and trial settings.

The oncological context has shifted. Neoadjuvant ADT is not standard before RP [7,8,9,11], but the rationale for earlier androgen-receptor blockade has strengthened with ARPI survival benefits [13,14,15], efficacy across hormone-sensitive settings [63,64,65], and expanding neoadjuvant programmes [66,67]. Yet this evidence remains dominated by pathological endpoints; NEAR, for example, reported no pathological complete response with 12-week apalutamide monotherapy, with functional outcomes underreported [17,18,19], precisely the gap that standardized digital monitoring can close.

Mechanistically, androgen deprivation reduces skeletal-muscle mass and performance and promotes sarcopenia [30,31,32], potentially impairing pelvic-floor and sphincter function; structured exercise can reverse ADT-related muscle loss, supporting a modifiable, muscle-mediated effect [68]. Notably, the continence signal emerged although all patients performed pre- and postoperative PFMT, pointing to a systemic rather than rehabilitation-related mechanism. Pharmacological timing offers a complementary explanation: the 6-month assessment fell during, or shortly after, ongoing postoperative hormonal exposure, whereas by 12 months, treatment was complete, compatible with an on-treatment, reversible effect, although this is unprovable with these data. The 12-month convergence suggests delay rather than permanent impairment, which nonetheless cannot be formally excluded on patient-reported measures. This pattern echoes the prior literature in which functional endpoints were underpowered or incomplete: ARNEO collected PROMs with limited follow-up and, lacking an upfront-surgery arm, could not quantify functional trade-offs versus immediate RP [69]; long-term quality-of-life analyses after conventional neoadjuvant therapy show nuanced, domain-specific patterns [12]. The recently reported PROTEUS trial demonstrated the oncological benefit of perioperative apalutamide plus ADT, showing a higher rate of pathological complete response or minimal residual disease (8.9% vs. 1.0%) and improved metastasis-free survival compared with placebo plus ADT [70], but both arms received ADT and surgery, so it does not isolate the functional impact of PHT versus upfront surgery; as intensification becomes practice-changing, characterizing functional trade-offs grows more important. SUGAR, which retains an upfront-surgery comparator, is positioned to provide such evidence [49]. The high 12-month continence in both groups matches contemporary RARP series and supports the technical adequacy of surgery [21,71,72,73].

Practically, this pathway is directly transferable: perioperative trials could embed digital continence assessment as a prespecified secondary endpoint with dual clinician/patient ascertainment at fixed anchors (6 weeks; 3, 6, and 12 months), daily symptom capture in the first postoperative month, baseline continence measurement with validated instruments [74], capture of the patient-estimated moment of recovery to reduce interval censoring [33], and prespecified missing-data handling. In routine care, the same infrastructure can flag deviant continence trajectories—for example, persistent pad use at 3–6 months during postoperative treatment—and trigger early pelvic-floor physiotherapy referral, for which randomized evidence shows benefit even in persistent incontinence [75].

Several limitations warrant emphasis. First, allocation was non-randomized, with confounding by indication; baseline characteristics were broadly comparable, but the mpMRI imbalance implies possible residual confounding. However, confounding by severity would bias the PHT group toward worse prognosis and would not readily explain a transient difference. Only 20 PHT patients contributed primary-endpoint data, precluding adjusted or stratified analyses. Second, the PHT exposure was small and heterogeneous, precluding regimen- or duration-specific inference; with 26 patients across several regimens, regimen-level reporting (counts, durations, completion) would yield uninformative subgroups and is deferred to larger prospective cohorts. The small sample also limits power for safety endpoints, whose null results are not evidence of equivalence. Third, social continence (0–1 pad/day) does not equate to strict pad-free continence, which was unavailable; the threshold was applied identically in both groups, and preoperative continence was ascertained clinically rather than with a validated instrument. Fourth, assessment was unblinded and follow-up incomplete, with differential completion of the clinician-reported primary endpoint (97% vs. 77%) remaining an acknowledged source of uncertainty; pain and analgesic endpoints are exploratory. Finally, the study was underpowered for uncommon events and long-term oncological endpoints; all findings are hypothesis-generating.

## 5. Conclusions

In this two-centre cohort of high-risk PCa patients undergoing RARP + LND, a routine-care digital monitoring pathway captured a delayed mid-term social-continence signal associated with PHT and lower clinician- and patient-reported continence at 6 months, converging by 12 months, compatible with a transient delay in recovery. Safety endpoints showed no statistically clear differences, though estimates were imprecise. Beyond the clinical signal, the study suggests that standardized digital monitoring is a practicable and informative method for capturing patient-centred recovery after RARP and supports incorporating it, with prespecified longitudinal continence endpoints, baseline measurement, and adjustment, into perioperative intensification trials.

## Figures and Tables

**Table 1:** Baseline characteristics.

Characteristic	Overall (*n* = 98)	SOC: Upfront RARP + LND (*n* = 72)	PHT + RARP + LND (*n* = 26)	*p*
Age, years (mean)	65.2	65.4	64.8	0.7
BMI, kg/m^2^ (mean)	25.6	25.1	26.5	0.1
Preoperative continence (no pads), *n* (%)	98 (100)	72 (100)	26 (100)	—
Pre- and postoperative PFMT, *n* (%)	98 (100)	72 (100)	26 (100)	—
ASA 2, *n* (%)	66 (82.5)	47 (87.0)	19 (73.1)	0.2
ASA 3, *n* (%)	14 (17.5)	7 (13.0)	7 (26.9)	
ASA, missing (*n*)	18	18	0	
Antiplatelet therapy, *n* (%)	13 (13.3)	9 (12.5)	4 (15.4)	0.8
Oral anticoagulation, *n* (%)	3 (3.1)	2 (2.8)	1 (3.8)	>0.9
PSA, ng/mL (mean)	14.6	14.0	16.2	0.5
Prostate volume, mL (mean)	46.5	48.2	41.9	0.1
cN1, *n* (%)	7 (7.1)	4 (5.6)	3 (11.5)	0.3
PI-RADS 3, *n* (%)	5 (5.1)	5 (7.0)	0 (0)	0.049
PI-RADS 4, *n* (%)	36 (36.7)	31 (43.1)	5 (19.2)	
PI-RADS 5, *n* (%)	57 (58.2)	36 (50.0)	21 (80.8)	
MRI stage iT2, *n* (%)	39 (39.8)	31 (43.1)	8 (30.8)	0.5
MRI stage iT3a, *n* (%)	45 (45.9)	31 (43.1)	14 (53.8)	
MRI stage iT3b, *n* (%)	14 (14.3)	10 (13.9)	4 (15.4)	
Biopsy GG 2, *n* (%)	8 (8.2)	4 (5.6)	4 (15.4)	0.1
Biopsy GG 3, *n* (%)	23 (23.5)	15 (20.8)	8 (30.8)	
Biopsy GG 4, *n* (%)	57 (58.2)	47 (65.3)	10 (38.5)	
Biopsy GG 5, *n* (%)	10 (10.2)	6 (8.3)	4 (15.4)	
Estimated blood loss, mL (mean)	162	170	140	0.3
Operative time, min (mean)	92	93	85	0.08
Nerve-sparing surgery, *n* (%)	17 (17.3)	11 (15.3)	6 (23.1)	0.4

Data are mean or *n* (%); percentages computed over non-missing observations. —, not applicable. PHT, perioperative hormonal therapy; PFMT, pelvic-floor muscle training; SOC (upfront RARP + LND) = standard of care; BMI, body mass index; ASA, American Society of Anesthesiologists physical-status class; PSA, prostate-specific antigen; MRI, magnetic resonance imaging; PI-RADS, Prostate Imaging Reporting and Data System; GG, grade group; cN1, clinical node-positive.

**Table 2:** Pathological and follow-up outcomes.

Outcome	Overall (*n* = 98)	SOC: Upfront RARP + LND (*n* = 72)	PHT + RARP + LND (*n* = 26)	*p*
RP GG 2, *n* (%)	10 (10.2)	8 (11.1)	2 (7.7)	0.5
RP GG 3, *n* (%)	39 (39.8)	28 (38.9)	11 (42.3)	
RP GG 4, *n* (%)	23 (23.5)	19 (26.4)	4 (15.4)	
RP GG 5, *n* (%)	26 (26.5)	17 (23.6)	9 (34.6)	
pT2, *n* (%)	35 (35.7)	26 (36.1)	9 (34.6)	0.9
pT3a, *n* (%)	42 (42.9)	30 (41.7)	12 (46.2)	
pT3b, *n* (%)	21 (21.4)	16 (22.2)	5 (19.2)	
pN1, *n* (%)	15 (15.3)	11 (15.3)	4 (15.4)	>0.9
Nodes removed (mean)	10.0	10.4	9.2	0.3
Positive nodes (mean)	2.7	3.3	1.3	0.14
Positive surgical margins, *n* (%)	27 (27.6)	22 (30.6)	5 (19.2)	0.3
Detectable PSA, *n* (%)	18 (18.4)	16 (22.2)	2 (7.7)	0.1
Biochemical recurrence, *n* (%)	33 (33.7)	26 (36.1)	7 (26.9)	0.4
Clinical recurrence, *n* (%)	11 (11.2)	10 (13.9)	1 (3.8)	0.16

Data are *n* (%) or mean. Recurrence data are descriptive; follow-up duration was not uniformly captured. PHT, perioperative hormonal therapy; SOC (upfront RARP + LND) = standard of care; RP, radical prostatectomy; GG, grade group; PSA, prostate-specific antigen.

**Table 3:** Postoperative recovery, social continence, and safety outcomes.

Outcome	SOC: Upfront RARP + LND (*n* = 72)	PHT + RARP + LND (*n* = 26)	Risk Difference, pp (95% CI)	*p*
Length of stay, days (mean)	0.72	0.77	—	0.7
Same-day discharge, *n* (%)	27 (37.5)	8 (30.8)	—	0.5
6-week social continence, clinician, *n*/*N* (%)	47/71 (66.2)	13/23 (56.5)	9.7 (−12 to 32)	0.4
6-week social continence, patient, *n*/*N* (%)	48/72 (66.7)	12/21 (57.1)	9.5 (−12 to 32)	0.4
6-month social continence, clinician, *n*/*N* (%)	61/70 (87.1)	13/20 (65.0)	22.1 (2.6 to 44.7)	0.02
6-month social continence, patient, *n*/*N* (%)	52/64 (81.3)	14/24 (58.3)	22.9 (2.4 to 43.9)	0.03
Daily pads, incontinent men (mean)	0.8	1.1	—	0.7
Incontinence QoL impact, VAS	2.3	3.4	—	0.6
12-month social continence, clinician, *n*/*N* (%)	63/65 (96.9)	19/20 (95.0)	1.9 (−7 to 21)	0.7
Any-grade complications, *n* (%)	14 (19.4)	8 (30.8)	−11.3 (−32 to 6)	0.2
Readmission, *n* (%)	4 (5.6)	3 (11.5)	—	0.2
Reoperation, *n* (%)	1 (1.4)	2 (7.7)	—	0.1
Unplanned visit, *n* (%)	3 (4.2)	4 (15.4)	—	0.06
Patient satisfaction, VAS	8.6	8.8	—	0.4

Data are mean, *n* (%), or *n*/*N* (%) over non-missing assessments. Social continence = 0–1 pad/day. Risk difference = Upfront RARP + LND (SOC) − PHT (percentage points), Newcombe 95% CI; positive values favour upfront surgery. —, not applicable. pp, percentage points. PHT, perioperative hormonal therapy; CI, confidence interval; QoL, quality of life; VAS, visual analogue scale (0–10).

**Table 4:** Postoperative pain and analgesic-use trajectories captured by digital monitoring.

Time Point	Upfront RARP + LND (*n* = 72)	PHT + RARP + LND (*n* = 26)	*p*
POD1—analgesic-free (%)	39	18	0.3
POD1—pain VAS, mean (SD)	4.1 (2.4)	3.8 (2.1)	0.6
POD3—analgesic-free (%)	49	14	0.06
POD3—pain VAS, mean (SD)	3.1 (1.5)	3.1 (1.8)	>0.9
POD7—analgesic-free (%)	88	57	0.004
POD7—pain VAS, mean (SD)	1.7 (1.3)	2.3 (2.2)	0.3
POD10—analgesic-free (%)	100	71	0.02
POD10—pain VAS, mean (SD)	0.5 (0.5)	1.7 (1.3)	<0.001

Analgesic-free = proportion reporting no analgesic use among patients responding that day (exploratory). Daily denominators varied with the number of patients responding each day; trajectories are therefore reported as percentages only and should be interpreted as exploratory. PHT, perioperative hormonal therapy; POD, postoperative day; VAS, visual analogue scale (0–10); SD, standard deviation.

## Data Availability

The data presented in this study are available on reasonable request from the corresponding author. The data are not publicly available owing to privacy restrictions.
